# Insulin-like growth factor-1 induces regulatory T cell-mediated suppression of allergic contact dermatitis in mice

**DOI:** 10.1242/dmm.015362

**Published:** 2014-08

**Authors:** Bjarki Johannesson, Susanne Sattler, Ekaterina Semenova, Saveria Pastore, Teresa M. Kennedy-Lydon, Robert D. Sampson, Michael D. Schneider, Nadia Rosenthal, Daniel Bilbao

**Affiliations:** 1 Mouse Biology Unit, European Molecular Biology Laboratory (EMBL), Monterotondo, 00015, Italy.; 2 National Heart and Lung Institute, Imperial College, London W12 0NN, UK.; 5 Laboratory of Experimental Immunology, Istituto Dermopatico Immacolata, IRCCS, Rome 00167, Italy.; 6 Australian Regenerative Medicine Institute, Monash University, Clayton, VIC 3800 Australia.

**Keywords:** Insulin-like growth factor-1, Atopic dermatitis, Contact hypersensitivity, Regulatory T cells, Treg

## Abstract

Allergic contact dermatitis (ACD) is triggered by an aberrant hyperinflammatory immune response to innocuous chemical compounds and ranks as the world’s most prevalent occupational skin condition. Although a variety of immune effector cells are activated during ACD, regulatory T (Treg) cells are crucial in controlling the resulting inflammation. Insulin-like growth factor-1 (IGF-1) regulates cell proliferation and differentiation and accelerates wound healing and regeneration in several organs including the skin. Recently IGF-1 has also been implicated in protection from autoimmune inflammation by expansion of Treg cells. Here, we demonstrate that ectopic expression of IGF-1 in mouse skin suppresses ACD in a Treg cell-specific manner, increasing the number of Foxp3^+^ Treg cells in the affected area and stimulating lymphocyte production of the anti-inflammatory cytokine interleukin 10. Similar therapeutic effects can be achieved with systemic or topical delivery of IGF-1, implicating this growth factor as a promising new therapeutic option for the treatment of ACD.

## INTRODUCTION

Allergic contact dermatitis (ACD) is a common inflammatory skin condition induced by exposure to frequently encountered environmental agents including metals, cosmetics, drugs and plant material. Clinical symptoms of ACD include itching with erythema, vesicles and blisters during acute phase, and cracks and fissures in the chronic phase (Usatine and Riojas, 2010). ACD can have a significant influence on the quality of life of affected individuals in addition to a considerable socio-economic impact (Kimber et al., 2002; Ale and Maibacht, 2010). Knowledge of the molecular mechanisms and the pathophysiology of ACD has mainly been derived from contact hypersensitivity (CHS) animal models in which skin inflammation is induced by painting the skin with haptens such as 2,4-dinitrofluorobenzene (DNFB) (Simonetta and Bourgeois, 2011). CHS develops in two steps: induction or sensitization and the subsequent elicitation phase. In the sensitization step, haptens penetrate the skin and induce an innate immune response that leads to the priming of hapten-specific T cells, causing sensitization so that any subsequent exposure to the initial hapten elicits a vigorous secondary immune response at the point of contact. In humans, this culminates in the cutaneous inflammatory reaction defined clinically as ACD (Kimber et al., 2002; Kaplan et al., 2012; Vocanson et al., 2009).

Regulatory T (Treg) cells are potent suppressors of inflammatory responses and crucial for the maintenance of immunological homeostasis and self-tolerance (Sakaguchi et al., 2010; Geiger and Tauro, 2012; Lehtimäki and Lahesmaa, 2013). Reduced Treg cell numbers or aberrant Treg cell functions have been implicated in a variety of hyperinflammatory conditions, and therapies that restore or increase Treg cell numbers have shown beneficial effects in experimental settings as well as in human patients (Jäger and Kuchroo, 2010; Huang and Sattler, 2011). Treg cells also play an important role in the control and resolution of the inflammatory response during CHS. Depletion of Treg cells causes enhanced and prolonged ear swelling (Tomura et al., 2010), whereas adoptive transfer of Treg cells can suppress immune cell infiltration and ear swelling, an effect mediated primarily by the release of the immunosuppressive cytokine interleukin (IL)-10 (Ring et al., 2009).

The peptide hormone insulin-like growth factor-1 (IGF-1) is an essential regulator of survival, growth and differentiation, accelerating wound healing and enhancing regenerative processes in a variety of organs and tissues, including the skin (Semenova et al., 2008). In line with a commonly seen cross-talk between the endocrine and the immune system, IGF-1 has also been shown to ameliorate autoimmune conditions (Liu et al., 1997; Lovett-Racke et al., 1998; Smith, 2010). Anguela and colleagues reported that IGF-1 was protective during autoimmune diabetes, correlating with an increased Treg cell percentage in the liver (Anguela et al., 2013), and we have recently demonstrated direct stimulation of local Treg cell proliferation by systemic IGF-1 therapy in multiple autoimmune disorders (D.B., B.J., N.R. et al., unpublished).

To test the hypothesis that IGF-1-mediated Treg cell expansion is also beneficial in non-autoimmune inflammatory conditions, we induced CHS in mice that either overexpressed transgenic IGF-1Ea propeptide in the skin (K14/IGF-1Ea) or were treated with recombinant human IGF-1 (rhIGF-1) protein by systemic or topical administration to assess therapeutic relevance. We document reduced inflammation through ear swelling, histology, cytokine expression and number of Treg cells and verify a direct effect of IGF-1 on Treg cells by conditional genetic deletion of the IGF-1 receptor (IGF-1R). The Treg cell-dependent clinical benefit of IGF-1 in this model offers both mechanistic insights into ACD and a clinically relevant strategy for therapeutic application.

**TRANSLATIONAL IMPACT**

**Clinical issue**

Allergic contact dermatitis (ACD) is an inflammatory skin condition caused by contact with common environmental agents to which the affected individual was sensitized during a previous exposure. ACD ranks as the world’s most prevalent occupational skin condition, has a considerable socio-economic impact and significantly affects the quality of life of affected individuals. Clinical symptoms include itching of the skin with erythema (redness), vesicles and blisters during the acute phase, and skin cracks and fissures in the chronic phase. Although the precise mechanisms underlying the pathology of ACD remain unclear, a variety of immune cells are known to be activated during ACD and the failure of a specific subset of immune cells (regulatory T cells) to control the resulting inflammation is a major contributing factor to the pathology. Recently, insulin-like growth factor-1 (IGF-1) has been implicated in conferring protection to other forms of hyper-inflammatory conditions by stimulation of regulatory T cells. The present study sought to determine whether IGF-1 is effective in suppressing symptoms of contact hypersensitivity (CHS), using a mouse model of ACD.

**Results**

To investigate the effect of IGF-1 on CHS symptoms, CHS was induced in transgenic mice ectopically expressing IGF-1 in skin cells. These mice showed a striking reduction in the inflammatory response and clinical CHS symptoms. Similar therapeutic effects could be achieved with either systemic delivery of IGF-1 by implanted osmotic minipumps or topical treatment of the affected skin area with hydrogel containing IGF-1. The suppression of inflammation and beneficial clinical outcome correlated with an increase in regulatory T cell numbers and elevated production of anti-inflammatory cytokines in the affected skin area. Most importantly, the severity of CHS symptoms in mice lacking the IGF-1 receptor specifically on regulatory T cells did not improve after IGF-1 treatment. This strongly indicates that the beneficial effects of IGF-1 are mediated by direct stimulation of regulatory T cells.

**Implications and future directions**

This study shows for the first time that IGF-1 directly stimulates the expansion of regulatory T cells and is able to suppress the pathological inflammatory reaction during contact hypersensitivity. The results of this study implicate this growth factor as a promising new therapeutic option for the treatment of ACD. In addition, due to its broad effect on regulatory T cell expansion, application of IGF-1 could also have therapeutic potential in patients with other acute inflammatory conditions, prompting further testing of its general efficacy in inflammatory disorders.

## RESULTS

### Overexpression of IGF-1Ea propeptide in the skin reduces the severity of contact hypersensitivity responses

**Fig. 1.**
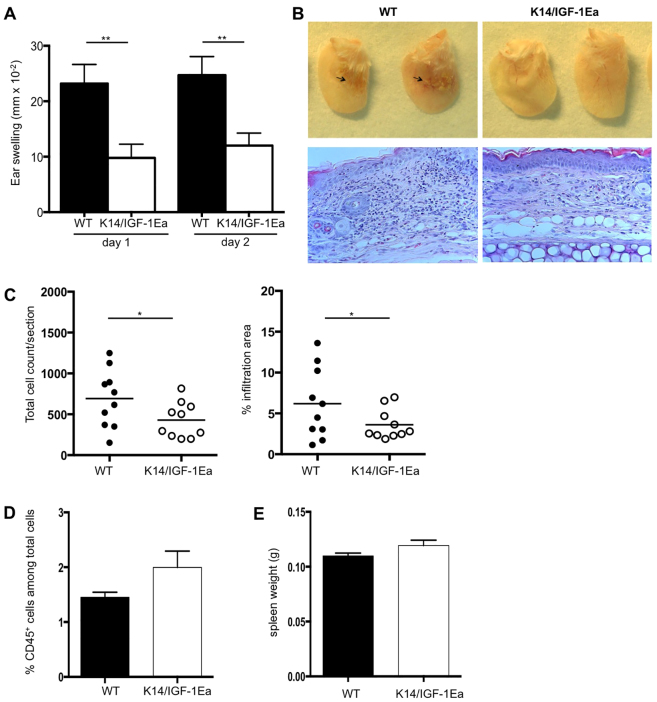
**Expression of transgenic IGF-1Ea propeptide in the skin reduces the severity of contact hypersensitivity responses.** (A) Inflammatory reaction (ear swelling) 24 and 48 hours after elicitation of a CHS response in K14/IGF-1Ea mice as compared with wild-type (WT) mice (*n*=14–16, FVB mouse strain). The experiment was repeated seven times by independent researchers and the graph depicts pooled data from two experiments. (B) Upper panels: macroscopic indications of inflammation (arrows) in wild-type and K14/IGF-1Ea ears 24 hours after elicitation. Lower panels: Immunohistological analysis of wild-type and K14/IGF-1Ea ears in ear cross-sections stained with H&E to visualize pathological changes and inflammatory infiltration. One representative staining of the indicated experimental groups is shown (*n*=10/group, repeated three times). (C) Quantification of pathology in H&E stained ear sections 24 hours after elicitation using the total number of nuclei per section (left panel) or area of infiltration (right panel). (D) Flow cytometric analysis of baseline resident CD45^+^ immune cells in the skin of K14/IGF-1Ea mice and wild-type controls. (E) Spleen weight of K14/IGF-1Ea mice and wild-type controls. *P<0.05, **P<0.01.

Previous studies investigating the role of IGF-1 in the immune system focused on the effects of systemic IGF-1 administration during autoimmune disease (Anguela et al., 2013; D.B., B.J., N.R. et al., unpublished). To study the effect of locally produced IGF-1 on tissue immune responses, we used the model of acute contact hypersensitivity in transgenic mice that express an IGF-1 propeptide (IGF-1Ea) ectopically in the skin (K14/IGF-1Ea mice) (Semenova et al., 2008), which improves wound healing and accelerates hair follicle formation and cycling. The E-peptide moieties of IGF-1 facilitate binding to the extracellular matrix, thereby controlling local IGF-1 bioavailability (Hede et al., 2012). As depicted in Fig. 1A–C, overexpression of IGF-1Ea in the skin significantly suppressed hypersensitivity responses. Macroscopic examination of ears and histological analysis of ear sections revealed protection from the CHS-induced swelling (Fig. 1A), macroscopic skin lesions (Fig. 1B, upper panels) and histopathological changes (Fig. 1B, lower panels). Immune cell infiltration was quantified by the total number of infiltrating immune cells (Fig. 1C, left panel) and the percentage of tissue area infiltrated with mono- and polymorphonuclear cells (Fig. 1C, right panel). To ensure that these effects were not due to a baseline change in the amount of CD45^+^ immune cells present in the skin and lymphoid organs of the K14/IGF-1Ea mice, CD45^+^ immune cells were isolated from the skin of naive unchallenged mice. No significant difference was observed in the frequency of CD45^+^ cells between K14/IGF-1Ea and control mice (Fig. 1D). Spleen weight as a measure of systemic immune cell proliferation was also comparable between age- and sex-matched mice of both K14/IGF-1Ea and wild-type controls (Fig. 1E). These results show that IGF-1Ea propeptide locally expressed in the skin can modulate immune cell function during acute immune responses to specifically suppress the hyperinflammatory response during CHS.

### Transgenic IGF-1Ea increases Foxp3 and IL-10 expression in skin T lymphocytes *in vivo*

**Fig. 2.**
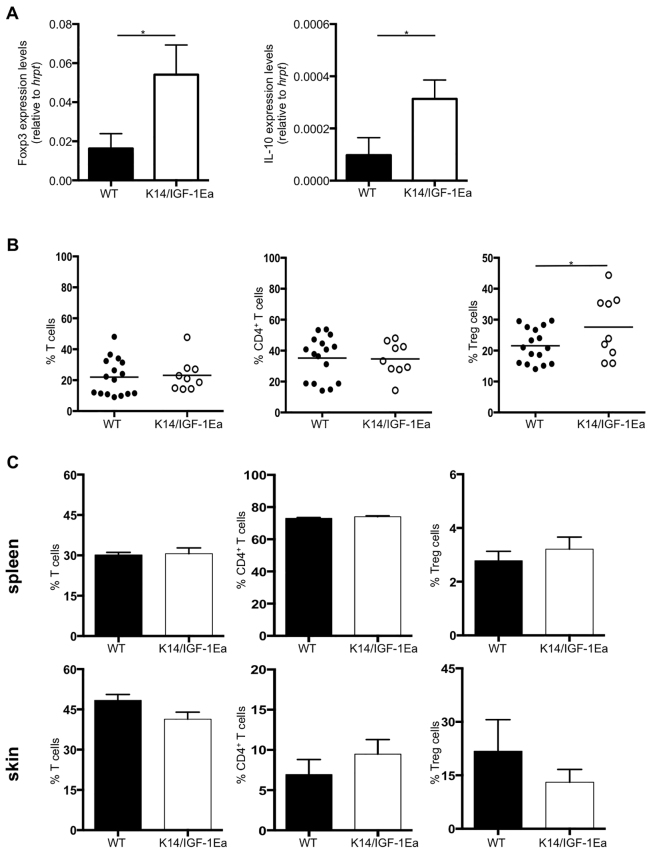
**Expression of transgenic IGF-1Ea propeptide in the skin increases local Treg cell numbers.** (A) Relative levels of mRNA expression of *Foxp3* and *IL-10* in CD4^+^ CD3^+^ CD45^+^ lymphocytes isolated from the skin of wild-type and K14/IGF-1Ea mice 48 hours after CHS treatment. Relative expression values were normalized to the expression of the housekeeping gene *hprt*. (B) Flow cytometric analysis of T cell populations: percentages of T cells (CD3^+^ among total CD45^+^ immune cells), CD4^+^ T cells (CD4^+^ among CD3^+^ T cells) and Treg cells (Foxp3^+^ among CD4^+^ cells) in the skin of wild-type and K14/IGF-1Ea mice 48 hours after contact CHS treatment. (C) Flow cytometric analysis of CD3^+^, CD4^+^ and Treg cell populations (CD3^+^ among total CD45^+^ immune cells, CD4^+^ among CD3^+^ T cells, and CD25^+^CD127^low^ among CD4^+^ cells) in the skin and spleen of unchallenged K14/IGF-1Ea mice and wild-type controls. *P<0.05.

To gain further insights into the mechanisms leading to IGF-1-mediated suppression of the acute contact hypersensitivity response, infiltrating CD4^+^ T lymphocytes were isolated from the skin of K14/IGF-1Ea and wild-type control mice and subjected to gene expression and flow cytometric analysis (supplementary material Fig. S1). After CHS elicitation following pre-optimised protocols (supplementary material Fig. S2), gene expression analysis of the immune suppressive cytokine IL-10 and the mouse Treg cell-specific transcription factor forkhead box P3 (Foxp3) (Sakaguchi et al., 2007) revealed increased mRNA levels of both factors in CD4^+^ T cells infiltrating the skin of K14/IGF-1Ea mice compared with wild-type control mice (Fig. 2A). To confirm that increased *Foxp3* mRNA levels represent an increased number of CD4^+^Foxp3^+^ Treg cells in the CHS-treated skin of K14/IGF-1Ea mice, T cell populations were further analysed directly using flow cytometry. This analysis revealed that the percentage of Foxp3^+^ Treg cells within the CD4^+^ T cell population in the skin of DNFB-treated mice was indeed significantly increased compared with untreated mice, whereas total T cell populations remained unchanged (Fig. 2B). We again excluded that this effect was due to baseline differences in the ratio between T cell populations in both spleen and skin, comparing wild-type and K14/IGF-1Ea mice (Fig. 2C). IL-10 protein levels were also slightly but significantly higher in cell culture supernatants of tissue-infiltrating CD45^+^ cells isolated from CHS challenged K14/IGF-1Ea mice (supplementary material Fig. S3A, left), whereas transforming growth factor (TGF)-β levels remained unchanged (supplementary material Fig. S3A, right). This might be due to other cell types producing TGF-β and masking the IGF-1 effect on Treg cells. Functionality of Treg cells in the skin after CHS challenge was further confirmed by IL-10 and TGF-β FACS staining of the Treg cell population (supplementary material Fig. S3B). Expression levels of both cytokines in Treg cells were comparable between wild-type and K14/IGF-1Ea mice, indicating that increased levels of IL-10 protein and mRNA in CD4^+^ and CD45^+^ cells and the beneficial effects of IGF-1 on CHS are due to the increase in Treg cell numbers, rather than a boost of the suppressive function of individual Treg cells.

There are several potential reasons for the increased number of Treg cells in the CHS-treated skin of K14/IGF-1Ea mice: local proliferation of skin-resident Treg cells, local *de novo* induction of Foxp3^+^ Treg cells, increased recruitment and infiltration of Treg cells, or combinations of these. To detect Treg cell proliferation in the skin, Treg cells from CHS-treated ear skin were stained with the proliferation marker Ki67. Further, all circulating blood cells were labelled *in situ* with carboxyfluorescein diacetate succinimidyl ester (CFSE) to determine Treg cell recruitment from the blood to the challenged skin. In addition to an increase in total Foxp3^+^ Treg cell numbers (supplementary material Fig. S4A), an increase in proliferating (Ki67^+^) Treg cells was detected in CHS-challenged ear skin of K14/IGF-1Ea mice as compared with wild-type mice (supplementary material Fig. S4B). However, very few Treg cells in the skin were positive for CFSE and no difference in the amount of CFSE^+^ Treg cells was detected between K14/IGF-1Ea mice and wild-type mice (supplementary material Fig. S4C). This suggests that local proliferation in response to locally produced IGF-1, rather than recruitment from the blood, accounts for the increased Treg cell numbers in the skin of K14/IGF-1Ea mice. Finally, *in vitro* rhIGF-1 treatment of isolated CD4^+^ T cells significantly increased the ratio of Treg cells to total CD4^+^ T cells, whereas Treg-depleted CD4^+^ T cell cultures failed to generate Foxp3^+^ Treg cells *de novo* (supplementary material Fig. S4D). Taken together, these data suggest that the IGF-1-mediated increase in Treg cells is due to an expansion of existing Treg cells rather than *de novo* induction. Thus, ectopic expression of IGF-1Ea propeptide in the skin increases the number of Foxp3^+^ Treg cells, probably by stimulating their proliferation locally, leading to a more immunosuppressive environment after the induction of an acute local inflammatory response in the skin.

### Ablation of IGF-1R in Treg cells abrogates the therapeutic effect of IGF-1

**Fig. 3.**
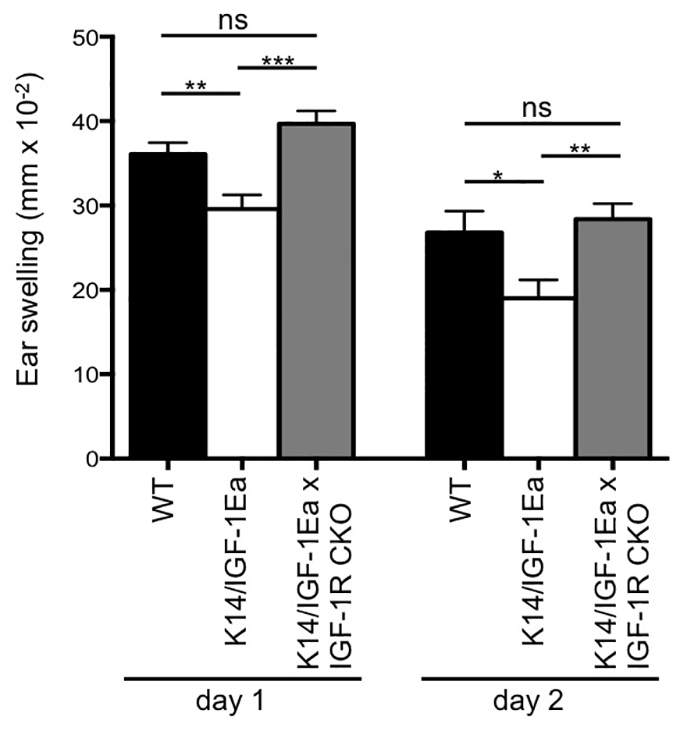
**Treg cell-specific deletion of the IGF-1R abrogates the beneficial effect of IGF-1.** Ear swelling response 24 and 48 hours after elicitation in mice with a Treg cell-specific deletion of the IGF-1R (IGF-1R CKO) crossed with K14/IGF-1Ea mice. Control mice were age- and sex-matched littermates on the same mixed genetic background of FVB × C57Bl/6xNOD. Wild-type (WT) mice carried neither the K14-IGF-1Ea transgene nor the IGF-1R CKO (K14-IGF-1Ea^wt/wt^, Foxp3Cre^wt/wt^, Igf1r^fl/fl^); K14-IGF-1Ea mice only lacked the IGF-1R CKO (K14-IGF-1Ea^tg/wt^, Foxp3Cre^wt/wt^ Igf1r^fl/fl^). The experiment was performed twice with comparable results and the graph depicts pooled data from both experiments (*n*=13–20). ns, not significant; *P<0.05, **P<0.01, ***P<0.001.

To investigate whether the observed suppressive effect of local IGF-1Ea propeptide on CHS in the skin was direct and dependent on Treg cells, K14/IGF-1Ea mice were crossed with mice harbouring a Treg cell-specific IGF-1R conditional deletion (IGF-1R CKO: Foxp3^Cre^ × Igf1r^fl/fl^). Although reproducible and statistically significant, suppressive effects of IGF-1Ea appeared reduced in these mice, potentially due to differences in genetic background that demanded the use of different CHS induction doses in order to yield comparable ear swelling responses (supplementary material Fig. S2). Interestingly, the significant therapeutic effect of IGF-1Ea overexpression on the severity of the contact hypersensitivity response was lost in IGF-1R CKO mice, which developed CHS ear swelling responses comparable with wild-type mice (Fig. 3). Thus, these results demonstrate that IGF-1R-mediated signalling in Treg cells is required for the immunosuppressive effect of IGF-1Ea during contact hypersensitivity, and confirm that this regulatory cell population is necessary for the observed IGF-1-dependent beneficial effect during this acute inflammatory response.

### Therapeutically relevant modes of IGF-1 delivery alleviate CHS induced ear swelling

**Fig. 4.**
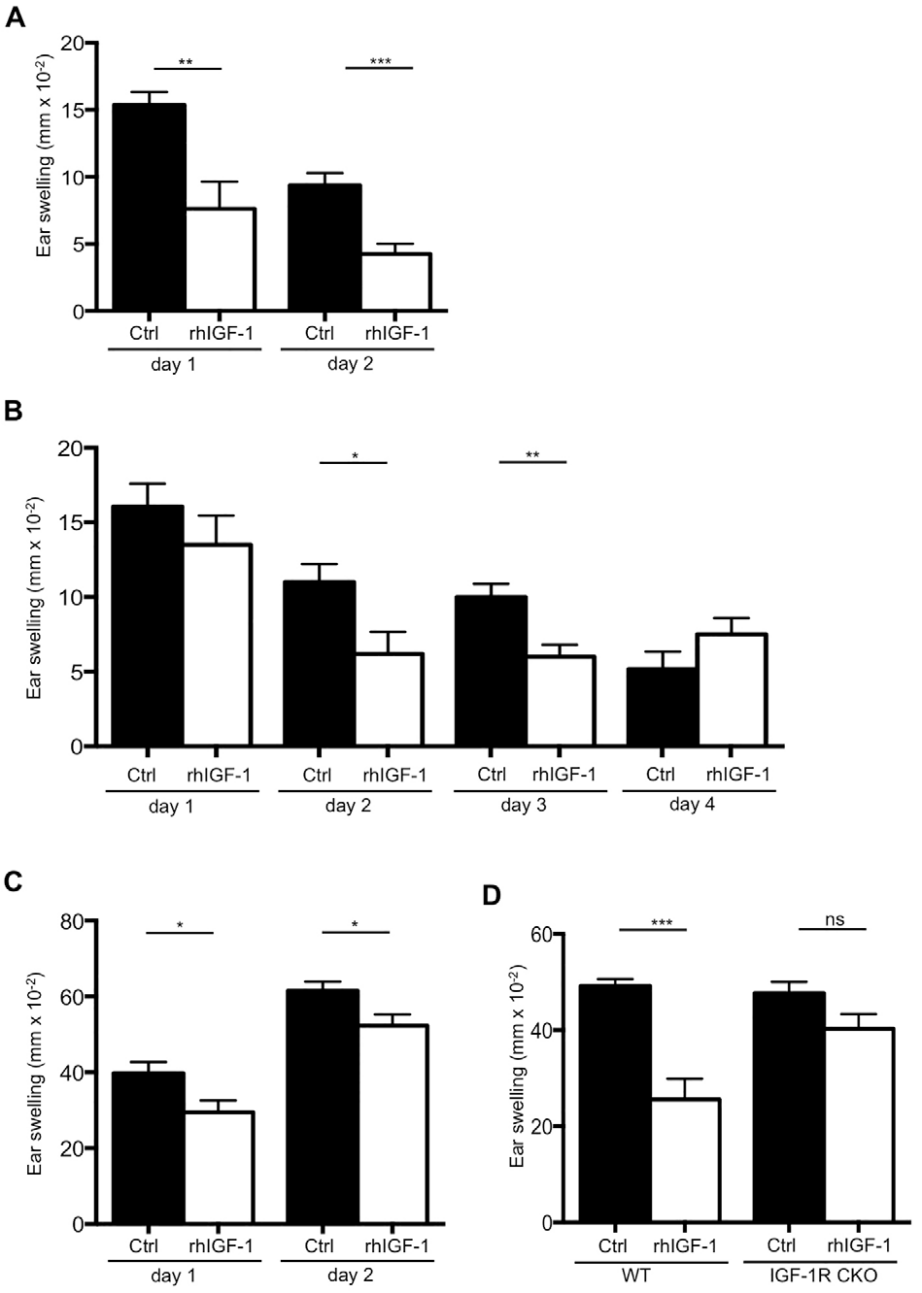
**Therapeutic delivery of rhIGF-1 reduces the severity of contact hypersensitivity responses.** (A) C57BL/6 mice were subcutaneously implanted with minipumps containing rhIGF-1. Control mice (Ctrl) were sham operated. One week after implantation, the mice were sensitized with DNFB and the CHS responses were elicited 5 days later (*n*=8). (B) Wild-type FVB mice were sensitized with DNFB and elicited 5 days later (*n*=16). At 3 days before and 2 days after elicitation, the mice were topically treated with 30 μl of rhIGF-1 hydrogel (100 μg/ml rhIGF-1). (C) At 4 days after first elicitation and 2 days after rhIGF-1, treatment was ceased and the mice were then re-elicited (*n*=8). (D) CHS was induced in wild-type (K14-IGF-1Ea^wt/wt^, Foxp3Cre^wt/wt^, Igf1r^fl/fl^) and IGF-1R CKO (K14-IGF-1Ea^tg/wt^, Foxp3Cre^wt/wt^ Igf1r^fl/fl^) mice. At 3 days before and 2 days after elicitation, the mice were topically treated with 30 μl of rhIGF-1 hydrogel (100 μg/ml rhIGF-1) on one ear; *n*=6 (WT Ctrl), 3 (IGF-1R CKO Ctrl), 7 (WT rhIGF-1), and 14 (IGF-1R CKO rhIGF-1). *P<0.05, **P<0.005, ***P<0.001; ns, not significant.

To demonstrate that IGF-1-mediated suppression of acute inflammatory skin conditions might also be applicable to clinical settings, therapeutically relevant protocols of IGF-1 delivery were investigated. Wild-type mice were subjected to the CHS protocol and evaluated after treatment with either systemic delivery of recombinant human IGF-1 (rhIGF-1) protein by subcutaneous implantation of minipumps or by topical application of a hydrogel containing IGF-1. As shown in Fig. 4, both delivery options were indeed beneficial during CHS because they significantly decreased inflammation in treated mice. Systemic delivery of rhIGF-1 resulted in a clear reduction in ear swelling, comparable to the effect observed after local IGF-1Ea overexpression in the skin (Fig. 4A). Topical treatment with hydrogel containing rhIGF-1, applied locally to the swollen skin area, caused inflammation to subside faster than in mice treated with hydrogel alone, although the initial difference in ear swelling was not significant between experimental groups (Fig. 4B). Topical treatment was ceased 2 days after elicitation when inflammation had subsided. Interestingly, when CHS was re-elicited for a second time 3 days later without further rhIGF-1 application, mice previously treated with rhIGF-1 hydrogel showed significantly less ear swelling and inflammation compared with untreated controls (Fig. 4C). Importantly, the therapeutic effect of IGF-1Ea-containing hydrogel was also lost in IGF-1R CKO mice, confirming that IGF-1Ea suppressed the inflammatory response during CHS by directly affecting Treg cells (Fig. 4D). These results clearly show that both systemic and local delivery options of IGF-1 have potent capacity to suppress acute skin inflammation during the course of the treatment, and that topical application might result in longer-lasting protective effects. Thus, the therapeutic benefit of IGF-1 in these models offers a clinically relevant strategy for the treatment of skin inflammation in ACD.

## DISCUSSION

In the current study we have demonstrated that IGF-1, delivered either as a transgenic propeptide or as recombinant processed protein, has immunosuppressive effects on acute inflammatory conditions, and that these effects are mediated by direct stimulation of Treg cells. Treg cells play a key role in maintaining and restoring self-tolerance and immunological homeostasis. They are well established as crucial protectors from autoimmunity, but they also facilitate the resolution of acute inflammatory responses and thereby prevent pathological inflammation (Lehtimäki and Lahesmaa, 2013). Treg cells are present in most peripheral organs and tissues including the skin and are crucially involved in maintaining tissue homeostasis under steady-state conditions (Clark et al., 2006; Sather et al., 2007). In addition, they are recruited to sites of tissue trauma or inflammatory insult, where they efficiently regulate and limit local inflammation (Siegmund et al., 2005; Yurchenko et al., 2006). During allergic contact hypersensitivity in a mouse model of allergic contact dermatitis, adoptive transfer of Treg cells has been shown to suppress immune cell infiltration and the resulting local swelling response (Ring et al., 2009) whereas depletion of Treg cells causes enhanced and prolonged skin inflammation (Tomura et al., 2010). In this study we show that transgenic IGF-1Ea propeptide or delivery of recombinant IGF-1 protein targets Treg cells, alleviating the acute allergic inflammation during contact hypersensitivity and reducing immune cell infiltration and ear swelling. IGF-1Ea propeptide also increased expression of the anti-inflammatory cytokine IL-10 and the Treg cell marker Foxp3, as well as the amount of infiltrating Treg cells in the affected skin area.

A number of autocrine or paracrine functions have been proposed for IGF-1 in the immune system. IGF-1 elicits a Th2-type response in cultured human T cells, stimulates T cell IL-10 production (Kooijman and Coppens, 2004) and augments plasma IL-10 concentrations in rats (Warzecha et al., 2003). It is therefore likely that local IGF-1 propeptides, produced predominantly by macrophages (Arkins et al., 1993), serve as a paracrine regulatory factor by inducing IL-10 production in T lymphocytes during inflammatory responses. Here, we extend these observations by defining the molecular basis of IGF-1 paracrine action on Treg cell stimulation *in vivo*. Because antibody-dependent depletion protocols (Anguela et al., 2013) cannot discriminate between direct and indirect effects and might suffer from lack of specificity and efficiency, we chose the more targeted approach of deleting the IGF-1R gene specifically on Treg cells, achieving a complete abrogation of the observed beneficial effects and confirming direct activation of Treg cells by IGF-1 in a disease context.

The results of this study elaborate on our previous findings on the beneficial action of IGF-1 in the context of heart repair, where cardiac-specific IGF-1Ea propeptide expression improved recovery from myocardial infarction (Santini et al., 2007). Further, myocardial transplantation of bone marrow mononuclear cells achieved improvements in left ventricular functional recovery after myocardial infarction, which was largely dependent on the presence of IL-10, as bone marrow from IL-10 knockout mice was not effective (Iekushi et al., 2012). Surprisingly, IL-10 deficiency of the transplanted cells did not affect infarct size, neutrophil accumulation and neovascularization, but rather was associated with decreased T lymphocyte accumulation, reactive hypertrophy and myocardial collagen deposition. Thus, in an acute injury context, comparable to the situation in an acute inflammatory insult, therapeutic IGF-1 also stimulates local T lymphocytes to secrete IL-10, limiting inflammation and promoting tissue repair.

The ability of IGF-1 to affect the ratio of pro- to anti-inflammatory cytokines in patients with an acute-phase response might also offer protection from multi-organ failure. Indeed, several trials have confirmed the safety of local and systemic application of IGF-1. Administration of IGF-1 to severely burned children resulted in significant improvement in outcome (Jeschke et al., 2000). Clinical improvement correlated with increased plasma levels of IL-10 and a reduction in pro-inflammatory cytokines (Jeschke et al., 2002), indicating that a least part of the beneficial effects of IGF-1 were due to its effects on the immune responses (Jeschke et al., 2002; Bozzola et al., 2003; Wolf et al., 2004).

Elucidating the function of IGF-1 in modulating immune responses directly through Treg cell-induced anti-inflammatory pathways in the skin suggests new possibilities for the treatment of allergic disorders. Therapeutically relevant delivery of IGF-1 for the treatment of allergic contact dermatitis could be achieved either by continuous systematic delivery of rhIGF-1 through osmotic minipumps or by topical application in hydrogel. Systemic delivery fully recapitulated the striking therapeutic effect of transgenic expression of IGF-1Ea propeptide in mouse skin. Topical application also reduced ear swelling in challenged mice, although with variable efficiency compared with systemic rhIGF-1 delivery. Removal of the hydrogel through exaggerated self-grooming, which was evident in treated mice, is a possible explanation for the lower efficacy in the topical model. Notably, mice treated with rhIGF-1 hydrogel showed a significantly reduced ear swelling in subsequent challenges with the same hapten, indicating that the protective effects of topically applied rhIGF-1 are sustained beyond its direct application period. This trait is of particular interest in the context of treating reoccurring ACD symptoms.

In summary, systemic or local rhIGF-1 delivery achieves pharmacological activation and expansion of endogenous Treg cells that is sufficient to convey protection, without the need for complex and time-consuming *ex vivo* Treg cell isolation, expansion and transfer protocols, thus holding obvious advantages over such technically challenging and expensive transplantation approaches. Because IGF-1 is an FDA-approved substance for replacement therapy in children (Ipsen Biopharmaceuticals, 2013) its application could have considerable therapeutic potential in patients with acute and/or persistent inflammatory conditions such as ACD, and clinical testing of its efficacy in allergic skin disorders could commence without delay.

## MATERIALS AND METHODS

### Mice

To delete the IGF-1R gene specifically in Treg cells, mice carrying loxP-modified IGF-1R alleles (Igf1r^fl/fl^, C57BL/6J; Jackson Laboratory, Bar Harbor, ME) (Dietrich et al., 2000) were crossed with Foxp3^Cre^ transgenic mice (NOD; Jackson Laboratory, Bar Harbor, ME) (Zhou et al., 2008). These Foxp3^Cre^Igf1r^fl/fl^ mice were then crossed with IGF-1Ea transgenic mice, in which IGF-1Ea expression is driven by the keratin 14 promoter in the skin (K14/IGF-1Ea, FVB) (Semenova et al., 2008). Foxp3EGFP mice (Wang et al., 2008) were kindly provided by Jian Guo Chai, Imperial College London. Mice used were 8–10 weeks old and control mice were age- and sex-matched littermates. Mice were housed in individually ventilated cages in temperature-controlled facilities on a 12-hour light/dark cycle on standard diet. All mouse procedures were approved by the European Molecular Biology Laboratory Monterotondo and the Imperial College London Ethical Committees and were in accordance with national and international regulations.

### Contact hypersensitivity

In K14/IGF-1Ea mice, which were on a FVB genetic background, contact hypersensitivity was induced by sensitizing mice on the shaved abdomen with 20 μl of 0.25% 2,4-dinitrofluorobenzene (DNFB) in a carrier mixture of 4:1 acetone:olive oil for two subsequent days. Elicitation was performed on day 5 with 10 μl 0.15% DNFB in 4: acetone:olive oil on each side of the right ear. For isolation of CD4^+^ cells for gene expression analysis, elicitation was performed on a 2-cm^2^ shaved area on the back torso of the mouse. All reagents were purchased from Sigma-Aldrich, Dorset, UK. Ear thickness was measured at 24 and 48 hours using a micrometer (Mitutoyo, Andover, UK). In line with previous reports showing reduced sensitivity of strongly pigmented mice to DNFB (Gaspari and Katz, 2001; Hayashi et al., 2009), C57Bl/6 and Foxp3^Cre^Igf1r^fl/fl^ × K14/IGF-1Ea mice, which were on a mixed genetic background (FVB × C57Bl/6xNOD), were treated with 0.5% DNFB for the sensitization and 0.15% for elicitation, a dose shown to yield swelling responses comparable to those in FVB mice (supplementary material Fig. S2A). Ear thickness was measured at 24 and 48 hours, as swelling peaks at 24 hours. In line with increased sensitivity to the DNFB treatment, the ear swelling in white K14/IGF-1Ea FVB mice took longer to resolve than in dark C57Bl/6 and mixed background mice. In white K14/IGF-1Ea FVB mice, ear swelling at 24 and 48 hours was comparable, but swelling peaked at 24 hours in C57Bl/6 mice and resolved quickly afterwards, so that differences between treatment groups were measurable only at early time points (supplementary material Fig. S2B). Ear swelling was determined by subtracting the ear thickness values of either untreated ears or the same ear before elicitation from the thickness values of treated ears. Initial experiments to ensure appropriate control measurements confirmed no difference in the ear thickness between left and right untreated ears (supplementary material Fig. S2C) nor between solvent (acetone:olive oil)-treated and untreated ears (supplementary material Fig. S2D). Ear swelling was the same when normalizing thickness measurements to either untreated ears or the same ear before elicitation (supplementary material Fig. S2E). Age and sex matched littermates, negative for transgene expression but on the same mixed genetic background, were used as experimental controls.

### Therapeutic rhIGF-1 delivery options

Systemic delivery of rhIGF-1 was achieved by continuous release of rhIGF-1 (Cambridge Biosciences, Milpitas, CA) at 0.25 μl/hour from an Alzet osmotic minipump (model #2004, Alzet Osmotic Pumps, Cupertino, CA) at a dose of 0.275 mg/kg/day. Minipumps were implanted in a skin pocket under the back skin under general anaesthesia. Topical treatment with rhIGF-1 was performed by applying 30 μl of hydrogel containing 100 μg/ml rhIGF-1 to one ear 3 days before and 2 days after elicitation of a CHS response.

### Immune cell isolation from the skin

Mouse external ear pinnae were harvested and separated into dorsal and ventral leaflets. To ensure standardized sampling and equal sample sizes, ears lobes were excised just above the cartilage area of the outer ear. For Fig. 2A, a 2 cm^2^ piece of challenged skin from the back torso was excised. Subcutaneous tissue was scraped off and the skin was finely minced and digested with 0.25% collagenase F (Sigma-Aldrich, Dorset, UK) for 30 minutes at 37°C. Digested tissue pieces were then mashed through a 70-μm sieve in order to generate a single cell suspension. The cell mixture was directly used for flow cytometric analysis of skin infiltrating cells or further processed for isolation of CD45^+^ or CD4^+^ cells by FACS sorting. For *in vitro* IGF-1 stimulation experiments, Foxp3EGFP mice were used and the CD4 population was FACS-sorted into CD4^+^GFP^+^ (total CD4) and CD4^+^GFP^−^ (Treg cell-depleted CD4) populations. FACS sorting was performed using FACS Aria or FACS Aria SORP (Becton Dickinson, Oxford, UK, 70 μm nozzle, 70 psi; >98% purity).

### Flow cytometric analysis

Antibodies against CD45 (clone 30-F11), CD3 (clone 17A2), CD4 (clone GK1.5), CD25 (clone PC61.5), CD127 (clone A7R34), Foxp3 (clone FJK-16F), Ki67 (clone 16A8), IL-10 (clone JES5-16E3) and TGF-β (clone TW7-16B4) were purchased from BioLegend (BioLegend, London, UK). Surface and intracellular stainings were performed according to the manufacturer’s protocol. For cytokine staining, cells were incubated for 4 hours with PMA/Ionomycin (Sigma-Aldrich, Dorset, UK) in the presence of GolgiStop (BD Biosciences, Oxford, UK) according to manufacturers’ instructions. To track potential Treg cell migration into the skin, blood cells were labelled *in situ* with CFSE by injecting mice with 2 μg CFSE/10 μl/g body weight over a 5-minute period via the tail vein (Becker et al., 2004). Samples were acquired using a BD LSRII (Becton Dickinson, Oxford, UK) and analysed using FlowJo (Treestar, Ashland, OR) software. The general gating strategy used for analysis is shown in supplementary material Fig. S1.

### Cell culture

Primary immune cells isolated from ear skin or spleen were cultured in RPMI-1640 medium (Hyclone, Logan, UT, USA) containing 2 mM L-glutamine, 10% fetal bovine serum (Sigma-Aldrich, Dorset, UK), 100 U/ml streptomycin and 100 U/ml penicillin (Hyclone, Logan, UT) at 37°C in a 5% CO_2_ atmosphere. For IGF-1 experiments, cells were stimulated with rhIGF-1 at concentrations of 25 and 100 ng/ml for 48 hours and processed further for flow cytometric staining of Foxp3 expression. To detect proliferation in some experiments, cells were labelled with eFluor780 (eBioscience, Hatfield, UK) prior to culture according to the manufacturer’s instructions. For intracellular cytokine staining, cells were stimulated for 4 hours with 50 ng/ml PMA and 500 ng/ml Ionomycin (Sigma-Aldrich, Dorset, UK) in the presence of GolgiStop (BD Biosciences, Oxford, UK) according to manufacturers’ instructions. For ELISA, cells were stimulated with 50 ng/ml PMA and 500 ng/ml Ionomycin for 24 hours.

### ELISA

CD45^+^ cells were FACS-sorted from CHS-treated skin and stimulated with PMA and Ionomycin for 24 hours. Cell culture supernatants were collected and used to detect secreted IL-10 and TGF-β. LEGEND MAX™ Mouse IL-10/TGF-β1 ELISA kits (BioLegend, London, UK) were used according to the manufacturer’s instructions.

### RNA isolation and quantitative PCR

RNA was isolated from CD45^+^CD3^+^CD4^+^ T cells obtained by FACS sorting from single cell suspensions of collagenase F-digested back skin using the RNeasy Mini Kit according to the manufacturer’s instructions (Qiagen, Manchester, UK). Total isolated RNA was used for cDNA synthesis using SuperScript first-strand synthesis system for RT-PCR (Life Technologies, Monza, Italy) according to manufacturer’s instructions. Quantitative RNA analysis was performed by real-time PCR using Taqman probes (Life Technologies, Monza, Italy) against IL-10 and Foxp3. Amounts of mRNA were normalized to the levels of the *hprt* gene, which was used as internal control.

### Histology

Ear pinnae from CHS-treated and untreated mice were excised and fixed in 4% formaldehyde overnight, dehydrated in an increasing gradient of ethanol and embedded in paraffin. Sections of 5 μm were cut and then dewaxed and rehydrated in an ethanol gradient. Sections were stained with hematoxylin and eosin (H&E). All reagents were purchased from Sigma-Aldrich (Sigma-Aldrich, Dorset, UK). Images were captured using a LMD7000 microscope (Leica Microsystems, Milton Keynes, UK) and quantified using the public domain software ImageJ (NIH; http://rsb.info.nih.gov) (Schneider et al., 2012).

### Statistics

Statistical analyses were performed using GraphPad Prism (GraphPad Software, La Jolla, CA) using a one- or two-tailed *t*-test as appropriate.

## Supplementary Material

Supplementary Material
