# Supplementary material for: Insulin-like growth factor-1 induces regulatory T cell-mediated suppression of allergic contact dermatitis in mice
Source: Dis Model Mech. 2014 Aug;7(8):977–85. doi: 10.1242/dmm.015362 (PMC4107326; doi:10.1242/dmm.015362)
Supplement: Supplementary Material [file supp_7_8_977__index.html]

Insulin-like growth factor-1 induces regulatory T cell-mediated suppression of allergic contact dermatitis in mice — Supplementary Material 

# Insulin-like growth factor-1 induces regulatory T cell-mediated suppression of allergic contact dermatitis in mice

## DMM015362 Supplementary Material

**Files in this Data Supplement:**

- **Supplementary Material**
