# Supplementary material for: Insulin-like growth factor-1 induces regulatory T cell-mediated suppression of allergic contact dermatitis in mice
Source: Dis Model Mech. 2014 Aug;7(8):977–85. doi: 10.1242/dmm.015362 (PMC4107326; doi:10.1242/dmm.015362)
Supplement: Supplementary Material [file supp_7.8.977_DMM015362.pdf]

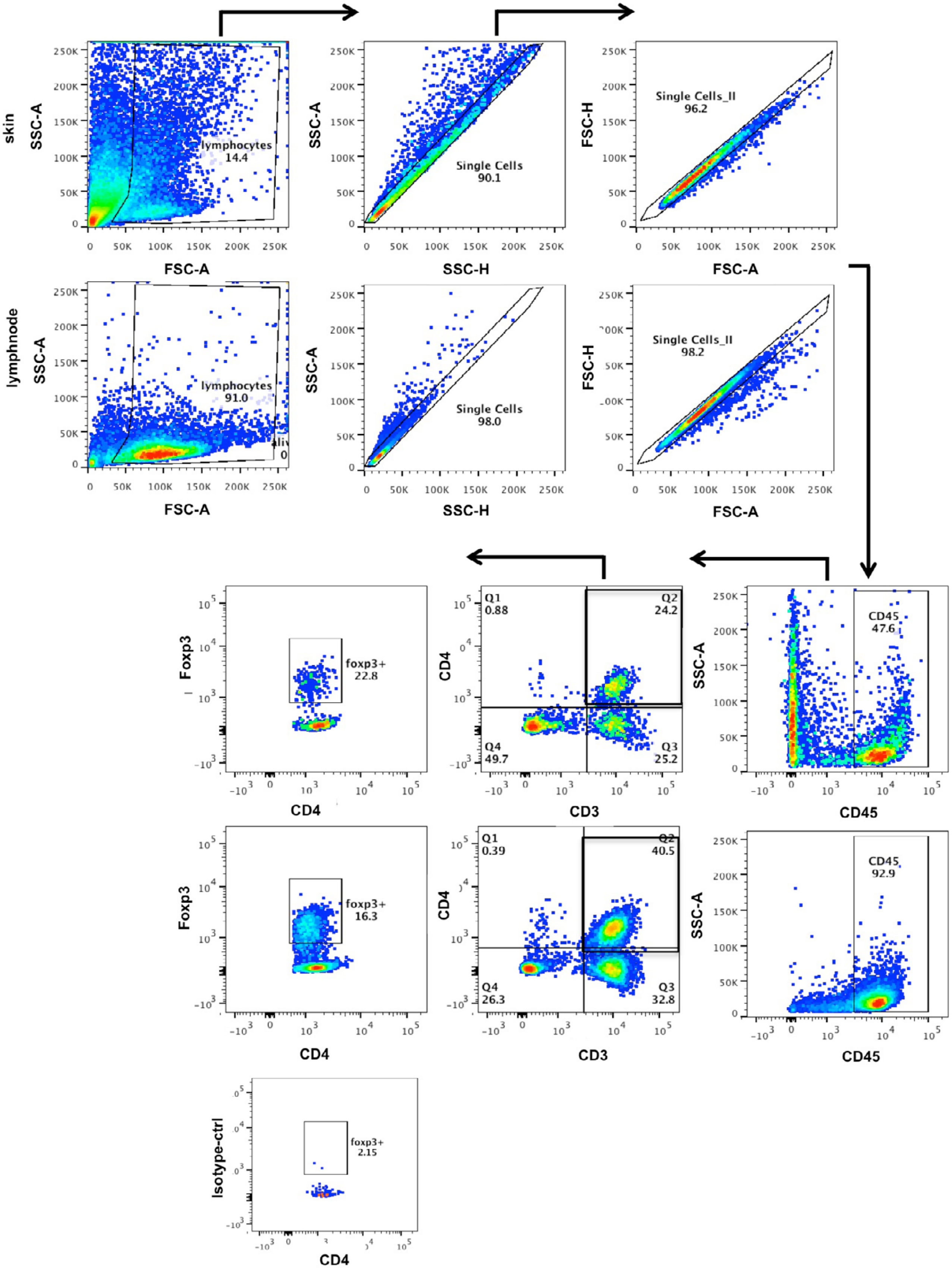

Fig. S1. Gating strategy for the quantification of Foxp3⁺ Treg in the skin. Cells were acquired as total cell population after digestion of the skin without additional prior purification. After gating for cells within the lymphocyte gate (chosen according to the size of the majority of cells in the lymph node control sample), cell duplicates were excluded by SSC-H/SSC-A and FSC-H/FSC-A gating. The $CD45^+$ cell population was obtained from the single cell population. Further gating included the $CD4^+$ population among $CD3^+$ T cells and the $Foxp3^+$ population among $CD3^+CD4^+$ T cells. To ensure specificity of intracellular Foxp3 staining, an isotype control sample was included.

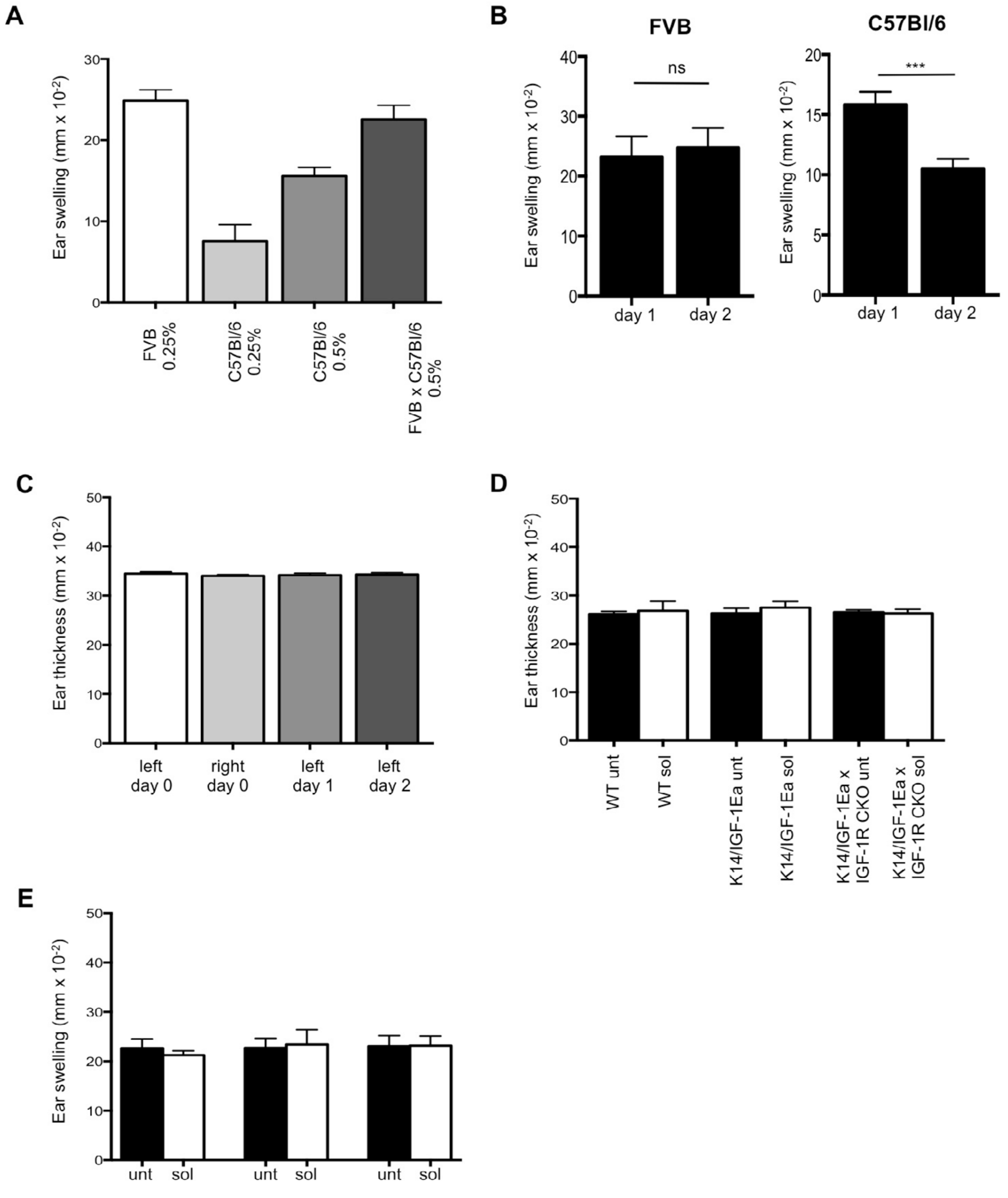

Fig. S2. Contact hypersensitivity treatment controls. (A) Ear swelling responses 24 hours after CHS elicitation in mice on FVB, C57Bl/6 and mixed (FVB x C57Bl/6xNOD) genetic backgrounds using either the ‘mild’ (0.25% DNFB) or the ‘harsh’ (0.5% DNFB) treatment protocol for CHS sensitization. (B) Ear swelling responses 24 and 48 hours after elicitation in wild type FVB and C57Bl/6 mice. (C) Comparison of ear thickness control measurements of right ears before treatment with DNFB (day 0) and untreated left ears over the course of the experiment (day 0-2). (D) Comparison of ear thickness control measurements of untreated (unt) and solvent (sol) treated ears in ‘WT′ (K14-IGF-1Ea$^{wt/wt}$, Foxp3Cre$^{wt/wt}$, Igf1r$^{fl/fl}$), ‘K14-IGF-1Ea’ (K14-IGF-1Ea$^{tg/wt}$, Foxp3Cre$^{wt/wt}$ Igf1r$^{fl/fl}$), ‘K14-IGF-1Ea x IGF-1R CKO′ (K14-IGF-1Ea$^{tg/wt}$, Foxp3Cre$^{tg/wt}$ Igf1r$^{fl/fl}$) mice on a mixed FVB x C57Bl/6xNOD genetic background. (E) Ear swelling responses calculated either by normalizing to untreated (unt) or solvent (sol) treated control measurements in ‘WT′ (K14-IGF-1Ea$^{wt/wt}$, Foxp3Cre$^{wt/wt}$, Igf1r$^{fl/fl}$) on a mixed FVB x C57Bl/6xNOD genetic background.

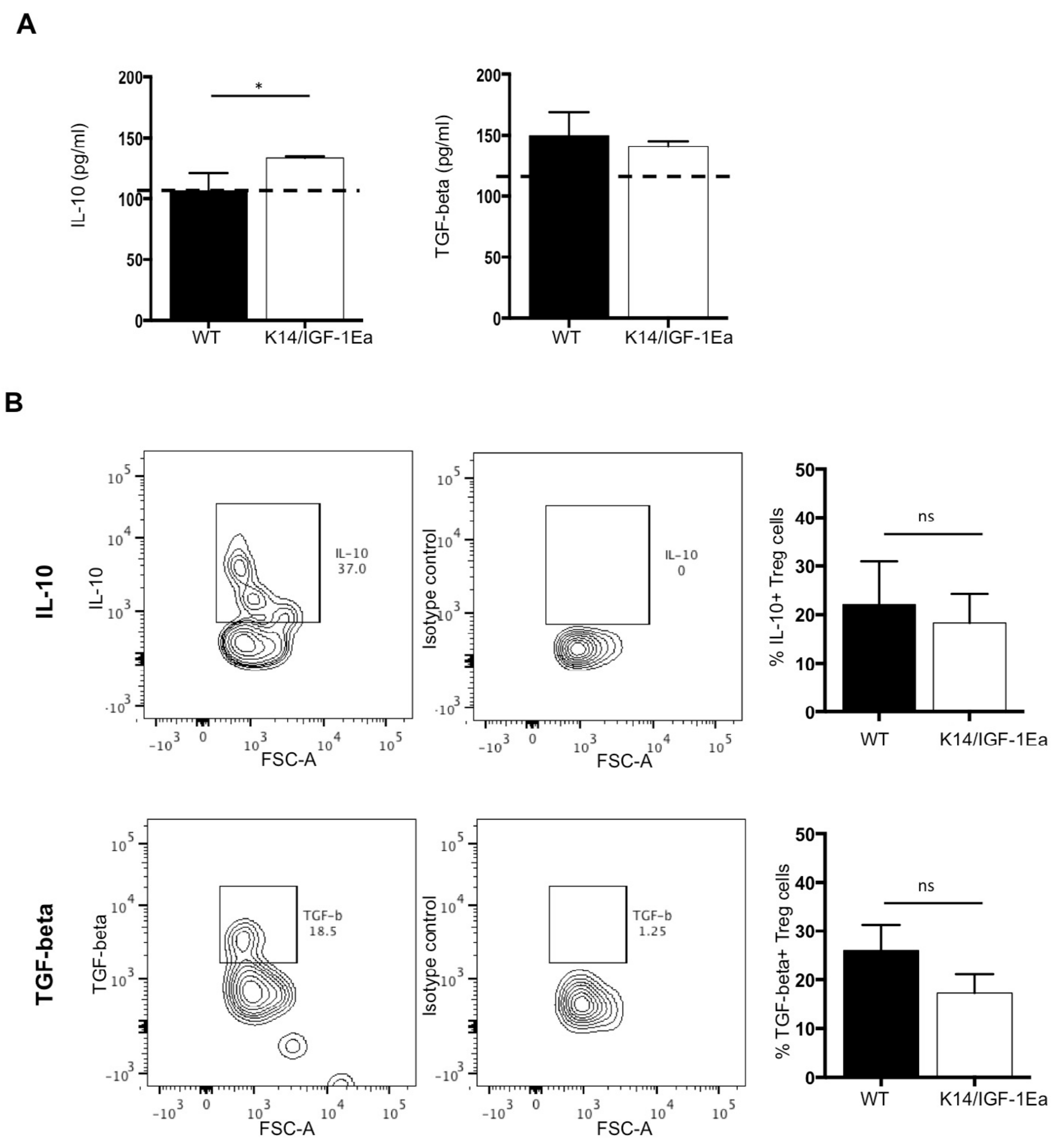

Fig. S3. IL-10 and TGF-beta expression of isolated Treg cells from CHS treated ears. (A) ELISA detecting IL-10 and TGF-beta levels in cell culture supernatant of CD45+ cells isolated from CHS treated ear skin of wildtype and K14/IGF-1Ea mice and stimulated for 24 hours with PMA/Ionomycin. (B) Flow cytometric analysis of IL-10 and TGF-beta expression in CD4+Foxp3+ Treg cells from CHS treated ear skin of wildtype and K14/IGF-1Ea mice and stimulated (as part of the total CD45+ cell population isolated from the skin) for 4 hours with PMA/Ionomycin/GolgiStop (left). Representative FACS blot showing IL-10 and TGF-beta staining (middle) compared to isotype control (right) staining in gated CD4+Foxp3+ Treg cells.

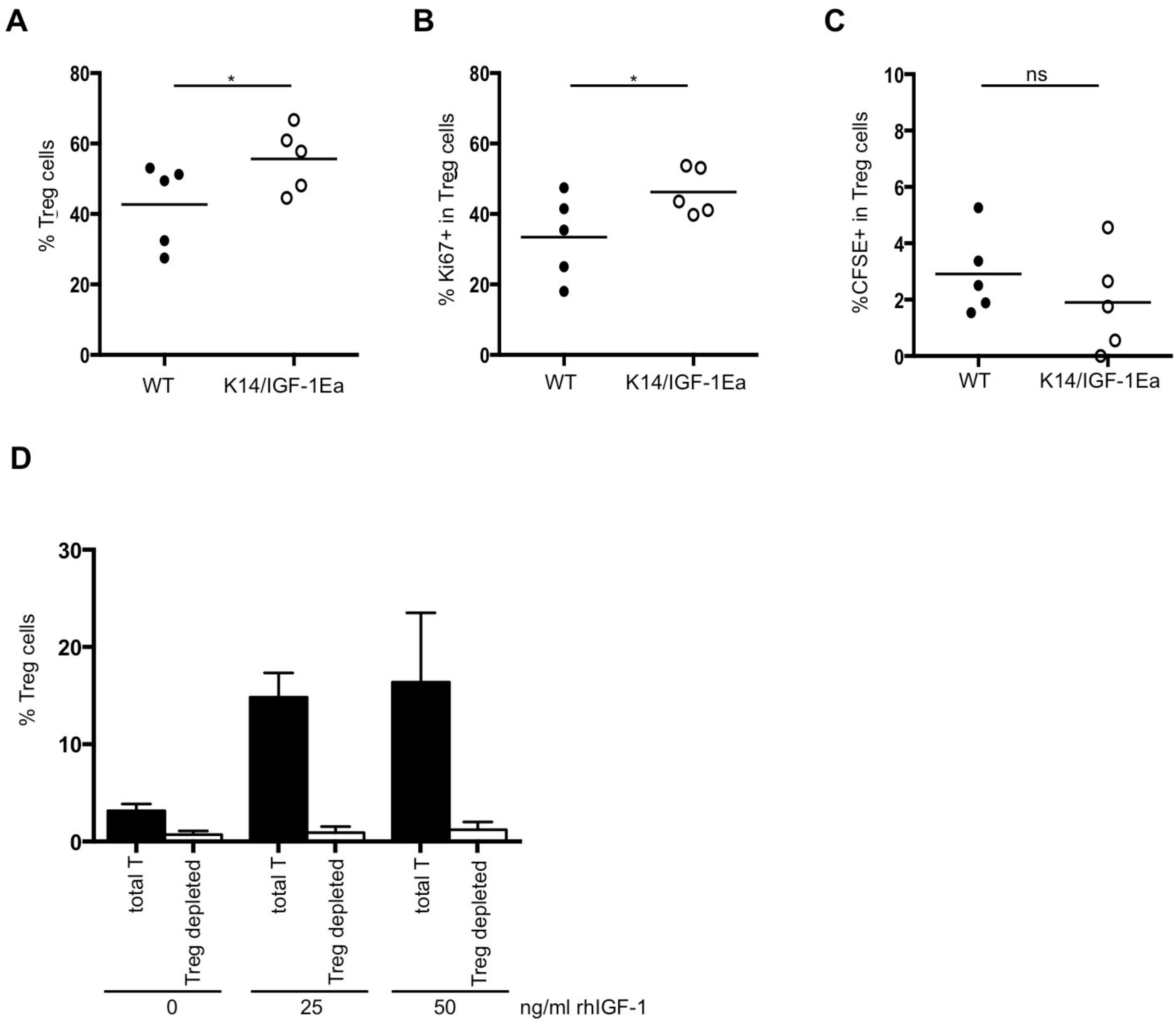

Fig. S4. Origin of Treg cells in CHS treated skin. Flow cytometric analysis of (A) % Treg cells (% $Foxp3^+$ among $CD4^+$ cells), (B) % Ki67+ cells among Treg cells and (C) %CFSE+ cells among Treg cells in the skin of wildtype and K14/IGF-1Ea mice 48 hours after contact CHS treatment. (D) % Treg cells (% $Foxp3^+$ among $CD4^+$ cells) after *in vitro* rhIGF-1 stimulation of total CD4 T cells and Treg cell-depleted CD4+ T cells.
